# Prizes and Honors

**Published:** 1900-05

**Authors:** 


### Prizes and Honors.

*Disease and Treatment (Senior).*

First prize—J. P. Foster, silver medal.
Second prize—C. D. McGilvray.
Third prize—W. K. Lewis.

Honors—W. P. Barnes, H. J. Daniels, D. F. De Wolf, B. K. Dow, F. E. Erwin, R. Frame, H. J. Hagerty, C. A. Hamilton, L. M. Holmes, O. Hartnagl, L. N. Jargo, E. E. Linton, J. Lucas, J. McFadyean, J. A. Rennie, A. C. Ramsay, B. A. Robinson, G. A. Shaw, G. O. Smith, E. J. Shirley, A. F. Smith, A. H. Stewart.

*Materia Medica (Senior).*

First prize—C. D. McGilvray.
Second prize—J. P. Foster.
Third prize—W. K. Lewis, A. C. Ramsay and A. F. Smith (equal).

Honors—F. J. Aldous, J. A. Campbell, D. S. De Wolf, H. J. Daniels, B. K. Dow, F. E. Erwin, R. Frame, C. A. Hamilton, O. Hartnagl, L. M. Holmes, L. N. Jargo, A. T. Lyster, E. E. Linton, J. Lucas, J. McFadyean, J. A. Rennie, B. A. Robinson, A. H. Stewart.

*Chemistry (Seniors).*

First prize—C. D. McGilvray.
Second prize—J.P. Foster and L. M. Holmes (equal).

Honors—D. T. De Wolf, R. Framer, H. J. Hagerty, C. A. Hamilton, L. N. Jargo, H. N. Jeffries, W. K. Lewis, A. T. Lyster, J. Lucas, E. E. Linton, A. C. Ramsay, R. T. Reece, B. A. Robinson, A. F. Smith, G. O. Smith, E. J. Shirley, A. H. Stewart.

*Pathology (Seniors).*

First prize—W. K. Lewis.
Second prize—C. D. McGilvray.
Third prize—A. F. Smith.

Honors—F. J. Aldous, J. A. Camplell, D. S. De Wolf, B. K. Dow, F. E. Erwin, J. P. Foster, C. A. Hamilton, L. M. Holmes, G. S. Jermyn, E. E. Linton, J. Lucas, J. McFadyean, B. A. Robinson, A. C. Ramsay, A. H. Stewart.

*Physiology (Senior).*

First prize—C. D. McGilvray.

Second prize—J. P. Foster.

Third prize—W. K. Lewis.

Honors—W. P. Barnes, B. K. Dow, C. A. Hamilton, L. M. Holmes, L. N. Jargo, A. C. Ramsay, B. A. Robinson, A. F. Smith, A. H. Stewart.

*Anatomy (Seniors).*

First prize—J. P. Foster.

Second prize—C. D. McGilvray.

Third prize—A. C. Ramsay.

Honors—W. P. Barnes, J. A. Campbell, A. L. Deal, D. S. De Wolf, B. K. Dow, F. E. Erwin, R. Frame, H. J. Hagerty, L. M. Holmes, L. Jargo, E. E. Linton, W. K. Lewis, J. Lucas, J. McFadyean, B. A. Robinson, A. F. Smith, A. H. Stewart, H. R. Stewart.

*Entozoa (Seniors).*

Prize—J. McFadyean.

Honors—H. Acres, F. J. Aldous, W. P. Barnes, B. K. Dow, F. E. Erwin, R. Frame, J. P. Foster, J. Freeman, C. A. Hamilton, H. N. Jeffries, L. N. Jargo, J. Lucas, A. T. Lyster, M. P. McLellan, C. D. McGilvray, C. H. McVeigh, J. A. Rennie, B. A. Robinson, G. A. Shaw, S. A. K. White.

*Dissected Specimens (Seniors).*

Gold medal, given by the Toronto Industrial Association—C. D. McGilvray.

Second prize—J. A. Campbell.

Third prize—F. E. Erwin.

*Best General Examination.*

Gold medal, given by the Ontario Veterinary Association—C. D. McGilvray.

Honors—B. K. Dow, F. E. Erwin, J. P. Foster, L. M. Holmes, W. K. Lewis, A. C. Ramsay, A.F. Smith.

